# Knowledge towards standard precautions among healthcare providers of hospitals in Amhara region, Ethiopia, 2017: a cross sectional study

**DOI:** 10.1186/s13690-020-00509-9

**Published:** 2020-12-01

**Authors:** Ayele Semachew Kasa, Worku Animaw Temesgen, Yinager Workineh, Tadesse Dagget Tesfaye, Sitotaw Kerie, Eden Amsalu, Solomon Emishaw Awoke

**Affiliations:** 1 Department of Adult Health Nursing, College of Medicine and Health Sciences, Bahir Dar University, Bahir Dar, Ethiopia; 2 Department of Pediatrics and Child Health Nursing, College of Medicine and Health Sciences, Bahir Dar University, Bahir Dar, Ethiopia; 3 Department of Emergency and Critical Care Nursing, College of Medicine and Health Sciences, Bahir Dar University, Bahir Dar, Ethiopia

**Keywords:** Infection prevention, Standard precautions, Knowledge, Healthcare workers, Amhara region, Ethiopia

## Abstract

**Background:**

Literatures revealed that healthcare-associated infections are still a great concern in many developing countries including in Ethiopia. Despite the development of detailed guidelines for infection control, they remain as a critical challenge for the public health sectors and the knowledge of standard precautions among healthcare workers in many developing countries is low and not properly applied. Hence, the present study tried to determine the level of knowledge about standard precautions among healthcare workers of Amhara region, Ethiopia.

**Methods:**

Institutional based cross-sectional study was conducted on a randomly selected public hospitals of Amhara region, Northwest Ethiopia from March 01–April 01/2017. A multistage sampling strategy was utilized to select 795 sampled healthcare workers. Data were collected using pretested self-administered questionnaire. The collected data entered using EpiData Version 3.1 statistical software and analyzed using SPSS version 20 statistical package. After using binary logistic regression, multivariable logistic regression analysis used to form the model. Variables which had statistically significant association with the outcome variable (*P* < 0.05) were identified as significant in the multivariable logistic regression analysis.

**Results:**

Almost half (49.2%) of the study participants were female healthcare workers. Three-fourth (74.3%) of the healthcare workers involved in the current study had good knowledge towards standard precautions. Good knowledge towards standard precautions refers to scoring correct responses to > 60% of knowledge items from the survey. Year of service (AOR: 0.27, 95% CI: 0.16 to 0.44), educational status (AOR: 1.7, 95% CI: 1.13 to 2.56) were among the predictor variables. In addition, physicians were 6.97 times more likely to be knowledgeable (AOR: 6.97, 95% CI 2.42 to 20.12) than laboratory technician/technology counterparts. Study participants working in medical, Gyn/obs, pediatrics wards, and OPD were about 2.23, 4.27, 2.81 and 2.52 times more likely to be knowledgeable than study participants working in surgical ward.

**Conclusions:**

Overall, the majority of healthcare workers had good knowledge of standard precautions. But variation in knowledge was detected across healthcare workers by hospital type and ward/units. This may help to design a solution by prioritizing the problem.

**Supplementary Information:**

The online version contains supplementary material available at 10.1186/s13690-020-00509-9.

## Background

Standard precautions (SPs) are intended for use to prevent the transmission of infection from one source to another. SPs are intended to protect healthcare providers, patients, and supporting staffs from nosocomial infections and occupational hazards [1, 2]. Healthcare-associated infections (HAIs) are the main cause of morbidity and mortality associated with clinical, diagnostic and therapeutic procedures [3, 4]. HAIs are not only a threat for healthcare workers (HCWs) but also a threat for service users and patients [5].

HAIs are pathogens that spread from one individual to others through a variety of ways. The most important spreading mechanism of these pathogens is via contaminated hands of the healthcare provider to the other healthcare provider, to patients or attendants of the patients. Fomites will also serve as a reservoir for potentially source of infectious agents like contaminated environmental surfaces, drugs, intravenous solutions or foodstuffs. The most important circumstances that a healthcare provider to be a risky group in any healthcare setting for HAIs are during direct patient care, instrument processing, surgical procedures, healthcare waste disposal, and processing patient care items [6, 7].

Different actions have been done tirelessly by the Federal Ministry of Health of Ethiopia to strengthen infection prevention measures. The measures mainly focused on bringing up-to-date information and practical interventions in the area of infection prevention [8]. Despite these efforts, in Ethiopia, the infection prevention activities are low [9, 10].

HAIs remain a critical challenge for the public health sectors in many developing countries [11]. Literatures revealed that HAIs are still a great concern in Ethiopian healthcare facilities [12, 13]. Despite the development of detailed guidelines for infection control, the knowledge of standard precautions among HCWs in many developing countries is low and not properly applied [11, 14]. A study revealed that SPs awareness has not been pronounced among healthcare workers, particularly in developing countries [15]. The Ethiopian Public Health Institute (EPHI) on services availability and readiness assessment informed that health workers must be able to work in a safe environment and must be provided with all the safety training need to carry out their duties. The assessment also revealed that there was a lack of knowledge on the proper utilization of SPs among different healthcare professionals [16].

Having adequate knowledge is a pre-requisite for implementing SPs in healthcare facilities. Poor knowledge about SPs among HCWs is the most common responsible reason for low adherence in implementing SPs in various healthcare facilities [17–19]. Hence, the present study tried to determine the level of knowledge among HCWs working in different healthcare facilities of Amhara region, Ethiopia towards SPs.

## Methods

The study was conducted in public hospitals in Amhara region from March 01–April 01/2017 using cross-sectional study design. Amhara region is one of the largest regions in Ethiopia found in the northwest direction. Currently, the region has more than 19 referral, zonal and district hospitals serving the populations of the region and neighboring residents. According to the 2015 Health Sector Development Plan-III (HSDP III) the Ethiopian health care tier system has different health facilities that consists of Referral hospital which serves approximately for 3.5 to 5.0 million, Zonal hospital serves for 1.0 to 1.5 million, and District hospital serves for 60,000 to 100,000 population [20].

The current study was carried out at eight randomly selected hospitals: two referral hospitals (Felege Hiwote and Gondar), four zonal hospitals (Debre Birhan, Debre Markos, Debre Tabor, and Dessie) and two district hospitals (Motta and Finote Selam).

HCWs involved in the provision of direct healthcare and those having contact with hospitals’ healthcare wastes were included in the study. Moreover, those study participants who are working in different inpatient and outpatient departments of the included hospitals were included.

### Sample size determination and sampling procedure

The sample size of the study was determined by using a single population proportion formula by taking (*P* = 37.7%) of the HCWs who had good knowledge towards standard precautions from a study done in Nigeria [21]. The 95% confidence interval, 0.5 margins of error, 10% none -response rate and a design effect of two were considered. Then, we reached at a final sample size of 795.

In order to select representative study participants, the total number of HCWs from each selected hospital were obtained. List of HCWs with their current working ward and profession was obtained from each hospital’s medical and matron offices. The total sample size was proportionally allocated in each hospital. Then from each hospital a study unit (HCWs) was selected using simple random sampling technique using their names’ list.

### Data collection

The data were collected using a structured self-administered questionnaire. The questionnaire was developed after reviewing different relevant literatures on the subject. It was divided into two parts. Part I: focused on socio-demographic characteristics and Part II: Questions to ascertain the level of knowledge towards standard precautions (Additional file 1).

### Data quality and control measures

The data collection tool developed after extensive literature search majorly from CDC and Ethiopian infection prevention guideline for health facilities [1, 22, 23]. First, the questionnaire was prepared in English and then translated to the local language (Amharic). Finally, the questionnaire was back-translated to the English language to confirm its consistency. To assess the reliability of the questionnaire, a pretest was conducted in 10% of the calculated sample sizes in Dangila hospital which was not included in the actual study.

Six junior nurses and three senior nurses were recruited as data collectors and supervisor respectively. Before the actual data collection period they received training about the questionnaire and how to approach the respondents. Completeness and clarity of the collected data were checked carefully on a regular basis. Furthermore, questionnaires with significant incompleteness were rejected from the whole analysis.

### Operational definitions

#### Scoring of knowledge

Knowledge was measured by a set of 11 questions. For every correct response, 1 point was given and 0 was given for an incorrect answer. Accordingly, knowledge scores ranged from 0 to 11.

#### Good knowledge to SPs

Those HCWs scored ≥60% out of knowledge assessing questions.

#### Poor knowledge to SPs

Those HCWs scored < 60% out of knowledge assessing questions. The knowledge score of the respondents was dichotomized as described above and this scoring system was also used in earlier studies [24, 25].

### Data processing and analysis procedure

Data entry was done by EPI info 3.5.1 and then transferred to SPSS version 20 statistical software for data cleaning, coding, and analysis. To explain the study population in relation to relevant variables, frequencies and summary statistics were done.

In order to determine the association between independent and dependent variables; multiple logistic regression analysis was performed. Variables with a *P*-value less than or equal to 0.20 in the bivariate model were included in the multivariable logistic regression model. Finally, a *P*-value of < 0.05 was considered as statistically significant.

## Result

Out of 795 sampled respondents, 765 agreed to participate in this study and from these, 742 participants completed all the questions without missing. As a result, 93.3% response rate was achieved. From the total six hospitals; 461 (62.1%) HCWs were from referral hospitals, 214 (28.8%) from Zonal hospitals and 67 (9.0%) were from district hospitals.

### Socio-demographic description of study participants

Of the total study participants, almost half (49.2%) were females. The majority (56.5%) were in the age range of 20–29 years. Regarding marital status, 51.6% were single whereas only 2% were widowed.

Concerning to educational status, 54.9% had bachelor degree whereas 4.3% were master’s degree holders. Professionally, the majority of study participants (55.9%) were nurses and 2.7% health officers. From all study participants, 40.3% had more than 6 years of work experience in the healthcare facilities. From the total study participants, 42.7% received training on SPs in the last 5 years. Almost one fifth (19%) of study participants worked at surgical wards and 9.6% at Pediatrics wards (Table 1).

**Table 1:** Socio-demographic characteristics of healthcare workers in Amhara region towards standard precaution, 2017

Variables	Frequency	Percent
Sex		
Male	377	50.8
Female	365	49.2
Age (in year)		
20–29	419	56.5
30–39	271	36.5
40–49	41	5.5
≥ 50	11	1.5
Marital status		
Single	383	51.6
Married	320	43.1
Divorced	24	3.2
Widowed	15	2.0
Educational status		
Diploma	303	40.8
Bachelor degree	407	54.9
Master’s degree	32	4.3
Religion		
Orthodox	594	80.1
Muslim	107	14.4
Protestant	33	4.4
Others^a^	8	1.1
Profession		
Nurse	415	55.9
Laboratory	108	14.6
Midwifery	104	14.0
Physician	95	12.8
Health officer	20	2.7
Work experience (in years)		
1–3	251	33.8
4–6	192	25.9
> 6	299	40.3
Training on SP in the last 5 year		
Yes	317	42.7
No	425	57.3
Hospital type		
Referral	461	62.1
Zonal	214	28.8
District	67	9
Ward		
Surgical	141	19.0
Medical	106	14.3
Gyn/obs	119	16.0
Pedi	71	9.6
OPD	95	12.8
Laboratory	108	14.6
Others^b^	102	13.7

^a^ = Catholic, No religion and ^b^ = Ophthalmic, orthopedics, Psychiatry, Emergency and Intensive Care Unit

*OPD* Out Patient Department, *Gyn/obs* Gynecology and obstetrics

### Overall knowledge of HCW towards SPs

In the current study, almost three-fourth (74.3%) of the study participants had good knowledge of standard precaution. Almost 85% of the study participants replied that adhering standard precautions protect HCWs getting infected from patients. Ninety two percent of the study participants replied that adhering standard precautions protect HCWs while handling infectious waste. More than 80% of HCWs replied that adhering standard precautions protect HCWs while handling sharp waste (Table 2).

**Table 2:** Healthcare workers knowledge regarding standard precautions in healthcare facilities of Amhara region, Northwest Ethiopia, 2017 (*n* = 742)

Items	Number (n)^a^	Percent (%)
All patients, healthcare workers and communities in healthcare facilities are at risk of health care related infection	582	78.4
Standard precautions should be applied to all patients regardless of their infectious status	621	83.7
Adhering standard precautions protect HCWs getting infected from patients	635	85.6
Adhering standard precautions protect patients getting infected from HCWs	530	71.4
Adhering standard precautions prevent mutual transfer of infection among patients	518	69.8
Adhering standard precautions protect HCWs while handling infectious waste	685	92.3
Adhering standard precautions protect HCWs while handling sharp waste	611	82.3
All patients/clients are potentially infectious irrespective of their diagnostic status?	525	70.8
Gloves should always be worn when have contact with any other body fluids except sweat?	529	71.3
Gown should always be worn during activities that are likely to generate splashes or sprays of blood, body fluids, secretions, or excretions.	547	73.7
A face mask, face shield, and/or goggles should be used if splashing of blood or body fluids might occur.	563	75.9

^a^Healthcare workers “Yes” response

### Factors associated with knowledge of study participants towards SPs

Candidate predictor variables from the bivariate regression model were entered into the multivariable logistic regression model. Among the nine variables entered into the bivariate model, one variable (training on SP) did not meet the criteria of significance (*p* > 0.2) to enter into the multivariable regression analysis. The multivariable logistic regression analysis showed that educational status, profession, service year, hospital type and ward type were shown to be significant predictors of knowledge towards SPs.

Participants with first-degree educational status were 1.7 times more likely to be knowledgeable than diploma holders (AOR: 1.7, 95% CI: 1.13 to 2.56). Regarding the professional category, nurses were 3.65 times more likely to be knowledgeable than their laboratory technician/technology counterparts (AOR: 3.65, 95% CI: 1.85 to7.18). Whereas, compared to laboratory technician/technology counterparts, physicians were 6.97 times more likely to be knowledgeable (AOR: 6.97, 95% CI 2.42 to 20.12).

Concerning to service year, study participants who served for more than 6 years were 73% less likely to be knowledgeable than study participants who served for less than 3 years (AOR: 0.27, 95% CI: 0.16 to 0.44. HCWs from zonal hospitals were 1.93 times more likely to be knowledgeable than study participants working at referral level hospitals (AOR: 1.93, 95% CI 1.18 to 3.14). Study participants working at medical, Gyn/obs, pediatrics wards, and OPD were about 2.23, 4.27, 2.81 and 2.52 times more likely to be knowledgeable than study participants working at surgical ward (AOR: 2.23, 95% CI: 1.17 to 4.26), (AOR: 4.27, 95% CI: 1.97 to 9.23), (AOR: 2.81, 95% CI: 1.31–6.03) and (AOR: 2.52, 95% CI: 1.25–5.07) respectively (Table 3).

**Table 3:** Factors associated with knowledge of healthcare workers in Amhara region towards standard precaution, 2017

Variables	Knowledge towards Standard precaution				COR (95% CI)	AOR (95% CI)
	Good knowledge		Poor knowledge			
	n	%	n	%		
Sex						
Male	294	78	83	22	1	1
Female	257	70.4	108	29.6	0.67(0.48–0.94)	0.76(0.51–1.12)
Age						
20–29	327	78	92	22	1	1
30–39	190	70.1	81	29.9	0.66(0.47–0.94)	0.97(0.64–1.48)
40–49	24	58.5	17	41.5	0.39(0.21–0.77)	1.05(0.47–2.34)
≥ 50	10	90.9	1	9.1	2.81(0.36–22.26)	6.1(0.71–52.4)
Marital status						
Single	301	78.6	82	21.4	1	1
Married	226	70.6	94	29.4	0.65(0.46–0.92)	0.86(0.57–1.29)
Divorced	15	62.5	9	37.5	0.45(0.19–1.08)	1.24(0.44–3.47)
Widowed	9	60	6	40	0.41(0.14–1.18)	0.65(0.17–2.46)
Educational status						
Diploma	212	70	91	30	1	1
First degree	323	79.4	84	20.6	1.65(1.17–2.33)	1.7(1.13–2.56)
Second degree and above	16	50	16	50.0	0.43(0.21–0.89)	0.8(0.35–2.21)
Profession						
Laboratory	60	55.6	48	44.4	1	1
Nurse	322	77.6	93	22.4	2.8(1.8–4.3)	3.65(1.85–7.18)
Midwifery	72	62.9	32	30.8	1.8(1.03–3.2)	1.4(0.56–3.48)
Health officer	9	45.0	11	55.0	0.66(0.25–1.71)	1.8(0.57–5.87)
Physician	88	92.6	7	7.4	10.1(4.3–23.7)	6.97(2.42–20.12)
Service year						
1–3	220	87.6	31	12.4	1	1
4–6	159	82.8	33	17.2	0.68(0.39–1.16)	0.8(0.45–1.42)
> 6	172	57.5	127	42.5	0.19(0.12–0.29)	0.27(0.16–0.44)
Training on SP						
Yes	230	72.6	87	27.4	1	§
No	321	75.5	104	24.5	1.17(0.84–1.63)	
Hospital						
Referral	318	69.0	143	31.0	1	1
Zonal	180	84.1	34	15.9	2.4(1.57–3.61)	1.93(1.18–3.14)
District	53	79.1	14	20.9	1.7(0.92–3.17)	1.67(0.82–3.45)
Ward						
Surgical	79	56.0	62	44.0	1	1
Medical	82	77.4	24	22.6	2.7(1.53–4.71)	2.23(1.17–4.26)
Gyn/obs	92	77.3	27	22.7	2.67(1.55–4.6)	4.27(1.97–9.23)
Pedi	57	80.3	14	19.7	3.19(1.63–6.21)	2.81(1.31–6.03)
OPD	78	82.1	17	17.9	3.6(1.94–6.7)	2.52(1.25–5.07)
Laboratory	77	71.3	31	28.7	1.95(1.14–3.32)	3.1(1.44–6.67)
Others^¥^	86	84.3	16	15.7	4.22(2.25–7.91)	3.8(1.89–7.88)

*OPD* Out Patient Department, § = Variables with *p* value of > 0.2 in bivariate analysis omitted from entering in to the multivariate model. Gyn/obs = Gynecology and obstetrics and ¥ = Ophthalmic, orthopedics, Psychiatry, Emergency and Intensive Care Unit

*COR* Crudes odds ratio, *AOR* Adjusted odds ratio, *CI* Confidence interval

## Discussion

Nosocomial infections and occupational hazards increase patients’ morbidity, mortality, length of hospital stay, and related treatment cost [26]. Hence, knowledge of standard precautions is important in preventing the occurrence of HAIs in healthcare settings. During patients’ care, it is thus of the highest importance for HCWs to have the knowledge of infection prevention and control measures [27]. This study aimed to determine the level of healthcare workers’ knowledge of standard precautions. The finding revealed that overall 74.3% of healthcare workers had good knowledge of SPs. This finding almost consistent with studies done in Zambia [28] and Nigeria [29] and Pakistan [14] in which 74.4, 74.6 and 73% of study participants respectively had good knowledge of infection prevention.

The finding revealed that study participants in the current study had good knowledge of standard precautions compared to some other studies. Studies were done in Nigeria [21, 30] Addis Ababa [31], West Arsi district [18] and Gondar University referral hospital [32] showed that 37.7, 65, 69, 53.7 and 57.4% of HCWs had good knowledge towards SPs respectively. This difference might be accounted for by the studies done in Nigeria included study participants from a single medical center. Whereas studies from Addis Ababa and Arsi focused not only hospitals but also on HCWs from health centers. Due to the above reasons, variation in the level of knowledge among HCWs might be result.

On the other hand, the overall knowledge of HCWs of the current study was lower than from studies conducted in different areas of Ethiopia. Studies from Dessie Referral Hospital [33], Bahir Dar City Administration [34] and Debre Markos Referral Hospital [35] revealed that 95.19, 84.2 and 84.7% of study participants had good knowledge towards SPs respectively. In addition, the current study also revealed a lower knowledge of SPs among HCWs than a study from the United Arab Emirates [36] that showed 97% of the respondents were knowledgeable. This discrepancy might be due to variations in sample size, access to training and differences in self-incitation in knowing about standard precautions. Moreover, receiving up-to-date information and training will result in an increment of knowledge of HCWs towards SPs. In the current study only less than half (42.7%) of study participants involved in SPs training programs. Such gaps might lower the overall knowledge of HCWs towards SPs in the current study.

The current study revealed that physicians were more knowledgeable than other professional groups. This finding was inconsistent with studies done in Tertiary Referral Center in North-Western Nigeria [37], Italy [38] and West Arsi [18] in which physicians were less knowledgeable than the rest of professional groups. Compared to other professional groups, a high amount of physicians were included in the current study. This might create the variation among the current study and other studies. In addition, by considering the total number of healthcare professionals in the healthcare system, physicians are more likely to participate in different pieces of training including infection prevention.

Many studies have shown that HCWs exhibited variable knowledge on standard precautions. This variation was accounted for by their years of experience [37]. Longer duration of professional experience to SPs shown to have an association with better knowledge towards standard precautions [39, 40]. Whereas the current study showed that work experience had an inverse association with overall knowledge of HCWs towards SPs. This finding also not consistent with a study at Debre Markos referral hospital that showed work experience significantly associated with knowledge [35]. This might be due to that study participants with greater work experiences will be at risk of being exposed to chronic fatigue at work [41]. This might lead them to experience workload, physical and psychological problems that may hinder them in participating to update themselves about SPs.

Though there were no studies reported whether variations accounted for by the hospital type, the current study revealed that study participants working in Zonal hospitals were knowledgeable. Study participants from Zonal level hospitals were almost two times more likely to be knowledgeable about SPs compared to HCWs from referral level hospitals. This variation might be focus was given for HCWs from Zonal level hospitals by the regional health bureau. Concerning working units, HCWs working in gynecology/obstetrics wards were more knowledgeable. This is obvious that HCWs from this ward frequently participate in different types of up-to-date training. This might help them to have good knowledge compared to other HCWs working in different wards/areas. In addition, HCWs at gynecology/obstetrics ward are more prone to expose for different body fluids. This might urge them to know about infection prevention strategies.

The Centers for Disease Control (CDC) has recommended that standard precautions be used in a better way when HCWs have adequate knowledge about them [42]. But Knowledge of standard precautions by HCWs may be influenced by different factors [43, 44]. These factors need to be addressed to implement standard precautions at all levels of healthcare facilities regardless of their staff composition, and institution type.

### Strength and limitation

The inclusion of different tier of hospitals and large sample sizes of professionals from different working settings/wards was the strength of this study. As a limitation, better evidence will be built if the study was conducted using observational study methods by incorporating HCWs practice in utilizing SPs. We didn’t assess on the safety measures taken from each healthcare institution. Multilevel model analysis to provide a clue on the cluster effect on such studies will have a paramount importance.

## Conclusion

This study has shown that the overall knowledge of HCWs toward standard precautions was good. However, variation in their knowledge level towards SPs was appreciated across hospital and ward types. For better management of infection prevention, healthcare managers and regional health bureau should assess healthcare providers’ knowledge towards standard precautions by hospital and ward types. This may lead to design a solution on how to develop a strategy in prioritizing an interventional act.

## Supplementary Information

**Additional file 1.** Questionnaire.

**Additional file 2.** Sampling strategy used to carry out the study.

## Data Availability

The datasets used and/or analyzed during the current study are available from the corresponding author on reasonable request.

## Abbreviations

CDC: Center for Communicable Disease Control
HAIs: Healthcare Associated Infections
HBV: Hepatitis B virus
HCV: Hepatitis C virus
HCWs: Health Care Workers
HIV: Human Immunodeficiency Virus
SPs: Standard Precautions
