# Supplementary material for: Knowledge towards standard precautions among healthcare providers of hospitals in Amhara region, Ethiopia, 2017: a cross sectional study
Source: Arch Public Health. 2020 Dec 1;78:127. doi: 10.1186/s13690-020-00509-9 (PMC7709327; doi:10.1186/s13690-020-00509-9)
Supplement: Supplementary file 1 — Additional file 1. Questionnaire. [file 13690_2020_509_MOESM1_ESM.docx]

**Additional file 1:**

**Questionnaire**

**English Version Consent form for participant**

We (Staffs of Bahir Dar University) are conducting a study to assess healthcare workers’ knowledge towards infection prevention in Hospitals of Amhara regional. I am going to give you the questionnaire which asks about some of your personal information and your knowledge on infection prevention measures. It will take about 20 minutes.

**Confidentiality**

Your records will be kept confidential. No information concerning you as individual will be passes to another individual or institutions. Your participation is voluntary and you have the right to not participate fully or partially.

**Subject Consent**

Do you agree to participate?

- Yes Give the questionnaire to continue
- No Thank him/her and proceed to other participant

Data collector ID: ______________

Date of data collection: ____/_____/_____

Time of beginning of the data collection: ______

**English Version Questionnaire**

| 1. General Information (To be filled by Data Collector) | | | |
| --- | --- | --- | --- |
| Qsn Code | Questions | Response | |
| 101 | Hospital Name | ---------------- | |
| 102 | Category of Hospital | 1. Referral 2. Zonal 3. District | |
| 103 | Ward/department name | --------------- | |
| 1. Personal Data (To be filled by Study Participant) | | | |
| 201 | Sex | | 1. Male 2. Female |
| 202 | Age in year | | _______ yrs |
| 203 | Marital Status | | 1. Single/never married 2. Married/cohabitant 3. Separated/ Divorced 4. Widowed 5. Other |
| 204 | Religion | | 1. Orthodox 2. Muslim 3. Protestant 4. Catholic 5. Other |
| 205 | Education Status | | 1. Read and write only 2. Grade 10/12 completed 3. College diploma 4. Fist degree 5. Medical Doctor (General Practitioner ) 6. Second Degree (MSc/MBA) 7. Medical Doctor Specialist 8. Other |
| 206 | Have you ever upgrade your educational status since your first employment | | 1. Yes 2. No |
| 207 | If yes for QSN No __ to what have you upgrade | | 1. To diploma 2. To BSc 3. To MSc 4. To other profession 5. Other (specify _____ |
| 208 | Profession | | 1. Laboratory Technician 2. Nurse 3. Midwife 4. Health Officer 5. Medical Doctor 6. Other (specify _________) 7. Rural |
| 209 | Year of work experience | | ______years _______months |
| 210 | Have you ever had training on standard precautions in the last 5 years? | | 1. Yes 2. No 3. Not sure |
| 1. Knowledge to infection prevention and control | | | |
| Q. Cod | Question | | Response |
| 301 | All patients, health care workers and communities in Health care facilities are at risk of health care related infection | | 1. Yes 2. No |
| 302 | Standard Precautions should be applied to all patients regardless of their infectious status | | 1. Yes 2. No |
| 303 | Adhering Standard Precautions Protect HCWs getting infected from patients | | 1. Yes 2. No |
| 304 | Adhering Standard Precautions Protect patients getting infected from HCWs | | 1. Yes 2. No |
| 305 | Adhering Standard Precautions Prevent mutual transfer of infection among patients | | 1. Yes 2. No |
| 306 | Adhering Standard Precautions Protect HCWs while handling infectious waste | | 1. Yes 2. No |
| 307 | Adhering Standard Precautions Protect HCWs while handling sharp waste | | 1. Yes 2. No |
| 308 | All patients/clients are potentially infectious irrespective of their diagnostic status? | | 1. Yes 2. No |
| 309 | When does hand washing is necessary? | | 1. On arrival at work 2. Before patient care 3. After patient care |
| 310 | Do gloves should be worn when have contact with blood? | | 1. Yes 2. No |
| 311 | Gloves should be worn when have contact with feces, urine? | | 1. Yes 2. No |
| 312 | Gloves should always be worn when have contact with any other body fluids except sweat? | | 1. Yes 2. No |
| 313 | Gown should always be worn during activities that are likely to generate splashes or sprays of blood, body fluids, secretions, or excretions. | | 1. Yes 2. No |
| 314 | A face mask, face shield, and/or goggles should be used if splashing of blood or body fluids might occur. | | 1. Yes 2. No |
| 315 | Syringes and needles should be disposed of as a single unit. | | 1. Yes 2. No |
| 316 | Used sharps should be carefully discarded into designated sharps containers at, the point of use. | | 1. Yes 2. No |
| 317 | Needles should not be re-capped, bent, broken or disassembled. | | 1. Yes 2. No |
