# Supplementary material for: Knowledge towards standard precautions among healthcare providers of hospitals in Amhara region, Ethiopia, 2017: a cross sectional study
Source: Arch Public Health. 2020 Dec 1;78:127. doi: 10.1186/s13690-020-00509-9 (PMC7709327; doi:10.1186/s13690-020-00509-9)
Supplement: Supplementary file 2 — Additional file 2. Sampling strategy used to carry out the study. [file 13690_2020_509_MOESM2_ESM.docx]

Sampling strategy used to carry out the study.

19 public Hospitals

Six hospitals were randomly selected

| F.Hiwot  N =342 | Gondar  N =362 | Debre Birhan  N =221 | Debre Markos  N =101 | Debretabor  N =140 | Dessie  N =262 | Mota  N =81 | Finoteselam  N =91 |
| --- | --- | --- | --- | --- | --- | --- | --- |

Proportional allocation to number of HCWs to each hospital

| F.Hiwot  n=170 | Gondar  n=180 | Debre Birhan  n=110 | Debre Markos  n=50 | Debretabor  n=70 | Dessie  n=130 | Mota  n=40 | Finoteselam  n=45 |
| --- | --- | --- | --- | --- | --- | --- | --- |

N= Total number of HCWs

HCWs were stratified by their profession and clustered in departments. The total sample size was proportionally allocated to each hospital. Then from each hospital a study unit (HCWs) was randomly selected using their names’ list.

n=sampled HCWs

795 Participants will involve in the study
